# A Novel Self-Temperature Compensation Method for Mode-Localized Accelerometers

**DOI:** 10.3390/mi13030437

**Published:** 2022-03-13

**Authors:** Pengcheng Cai, Xingyin Xiong, Kunfeng Wang, Liangbo Ma, Zheng Wang, Yunfei Liu, Xudong Zou

**Affiliations:** 1 State Key Laboratory of Transducer Technology, Aerospace Information Research Institute, Chinese Academy of Sciences, Beijing 100190, China; caipengcheng16@mails.ucas.ac.cn (P.C.); wangkunfeng17@mails.ucas.ac.cn (K.W.); maliangbo19@mails.ucas.ac.cn (L.M.); liuyunfei17@mails.ucas.ac.cn (Y.L.); 2 School of Electronic, Electrical and Communication Engineering, University of Chinese Academy of Sciences, Beijing 100049, China; 3 QiLu Aerospace Information Research Institute, Jinan 250101, China; wangzheng02@aircas.cn

**Keywords:** 2-DoF, mode-localized accelerometer, compensation

## Abstract

Mode-localized sensing paradigms applied to accelerometers have recently become popular research subjects. However, the output of mode-localized accelerometers is influenced by environment temperature due to the difference in the thermal properties of the coupling resonators and the temperature dependence of coupling stiffness. To improve the performance of mode-localized accelerometers against temperature, we proposed an in situ self-temperature compensation method by utilizing the resonant frequency besides of amplitude ratios, which can be implied online. Experimental results showed that there were nearly 79-times and 87-times improvement in zeros bias and scale factor, respectively.

## 1. Introduction

MEMS (Microelectromechanical systems) accelerometers have the advantages of small size, light weight, low power consumption and low cost [1]; thus, they are widely used in the fields of inertial navigation, medical consumer electronics and automotives [2,3]. Among various kinds of MEMS accelerometers, silicon resonant accelerometers are promising for high sensitivity, large linear range, low bias instability and so on [4,5,6,7]. 

Over the past few years, the mode-localized sensing paradigm based on weakly coupled resonators (WCRs) has been researched and applied to various kinds of sensors including accelerometers for its ultra-high sensitivity [8,9,10,11,12,13,14] and the suppression of common mode noises [15,16,17,18,19] with the output metric of the amplitude ratio. In a sensor using WCRs, weaker coupling strength means higher sensitivity. Some works have made efforts to achieve lower coupling stiffness, among which the electrically stiffness coupling and mechanical coupling structure are widely used. Compared with the scheme of electrical stiffness coupling, coupling structures lack the noise from a coupling voltage. As there is a difference in the properties of the coupled resonators, mode-localized accelerometers still suffer from the influence of temperature since there would be thermal perturbation as temperature fluctuates. Furthermore, as the Young’s modulus of the silicon material is very sensitive to temperature, mechanical coupling stiffness is temperature-dependent, making the temperature performance worse. To improve the temperature performance of MEMS accelerometers, some methods were proposed including active temperature control [20,21,22,23], temperature compensation [24,25,26] and less temperature sensitivity structure [27]. The active temperature control scheme requires complex temperature control systems and higher power consumption. The scheme of reducing the temperature sensitivity by structure design can only achieve a limited improvement. Therefore, a passive temperature compensation scheme is widely used for its simplicity. One of the passive temperature compensation methods is to make a compensated result with the output of the accelerometer and the temperature captured by an additional thermometer or temperature sensitive element. Another way is to implement self-temperature compensation by the two outputs from a differential structure of resonant accelerometer [28,29]. As there is no need for additional temperature measurement in the second way, the problem of thermal lag between the thermometer and the accelerometer is alleviated. However, this scheme of self-temperature compensation proposed for differential structure is not in situ exactly, as there is distance between the differential resonators. Furthermore, there have been less studies on the temperature compensation for mode-localized accelerometers.

In this article, we proposed a self-temperature compensation method which is an in situ compensation way for a two degree-of-freedom (2-DoF) mode-localized mechanically coupled accelerometer. To the best of our knowledge, this is the first work proposing a in situ self- temperature compensation method for 2-DoF mode-localized accelerometers by using amplitude ratios and resonant frequency together. A neural network was trained to study the relationship between the parameters with amplitude ratios and resonant frequency under different temperature. With a real-time measurement on resonant frequency besides of amplitude ratio, the proposed method for a 2-DoF mode-localized accelerometer can be applied online.

## 2. Two-DoF WCRs Accelerometer

**Figure 1:**
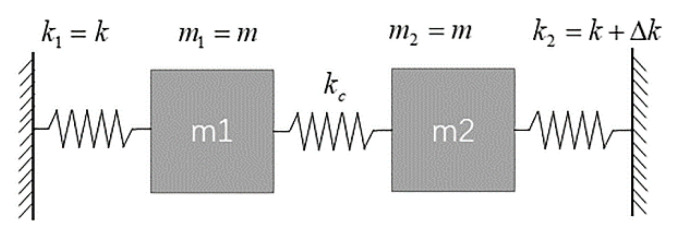
Mass–spring–damper model of a 2-DoF WCRs with external perturbation.

A simplified model of mode localized accelerometer based on a 2-DoF weakly coupled resonator is shown in Figure 1. Ideally the mass and the stiffness of the two resonators are initially symmetric: i.e., $m_{1}=m_{2}=m$, $k_{1}=k$, $k_{2}=k+\Delta k$, where $\;m_{1}$, $m_{2}$ represent the effective mass of each resonator and $k_{1}$, $k_{2}$ represent the effective stiffness of each resonator while Δk represents the stiffness perturbation, $k_{c}$ represents the coupling stiffness.

Without considering damping terms and driving force the motion of the system can be formulated as

(1) $$\left[\begin{array}{cc} m & 0\\ 0 & m \end{array}\right]\left[\begin{array}{c} {\ddot{x}}_{1}\\ {\ddot{x}}_{2} \end{array}\right]+\left[\begin{array}{cc} k+k_{c} & -k_{c}\\ -k_{c} & k+k_{c}+\Delta k \end{array}\right]\left[\begin{array}{c} x_{1}\\ x_{2} \end{array}\right]=\left[\begin{array}{c} 0\\ 0 \end{array}\right]$$

where $x_{1}$ and $x_{2}$ are the displacements of the two masses. With using the resonant frequency of first mode without structural perturbation $\omega_{0}=\sqrt{k/m}$, the resonant radian frequencies $\omega_{i}$ (i = 1, 2) and amplitude ratios $u_{i}$ (i = 1, 2) of the two modes can be derived as:

(2) $$\omega_{i}=\left(\sqrt{1+\frac{1}{k}\left(k_{c}+\frac{\Delta k}{2}\mp \frac{1}{2}\sqrt{\Delta k^{2}+4k_{c}^{2}}\right)}\right)\omega_{0}$$

(3) $$u_{i-12}=\frac{x_{i1}}{x_{i2}}=\frac{\Delta k\pm \sqrt{\Delta k^{2}+4k_{c}^{2}}}{2k_{c}}$$

(4) $$u_{i-21}=\frac{x_{i2}}{x_{i1}}=-\frac{\Delta k\mp \sqrt{\Delta k^{2}+4k_{c}^{2}}}{2k_{c}}$$

As the perturbation varies, the amplitude ratio $u_{i-12}$ in the first vibrational mode (i = 1) goes from good linearity to strong nonlinearity, while he amplitude ratio $u_{i-12}$ in the second vibrational mode (i = 2) is to the opposite. This trend of amplitude ratios $u_{i-21}$ (*i* = 1, 2) is opposite to the amplitude ratio $u_{i-12}$ (i = 1, 2). Work [30] proposed a method to enhance the linearity of WCRs by using output metric based on the subtraction of reciprocal amplitude ratios. This method can be implemented by simple calculation of the amplitude ratios from one WCRs. The method can be formulated as (i = 1, 2):

(5) $$u_{oi}=u_{i-12}-u_{i-21}=\frac{x_{i1}}{x_{i2}}-\frac{x_{i2}}{x_{i1}}=\frac{\Delta k}{k_{c}}$$

where $u_{oi}$ (i = 1, 2) is the linearity-enhanced output metric. By using linearity-enhanced output metric, a larger linear range can be achieved. 

**Figure 2:**
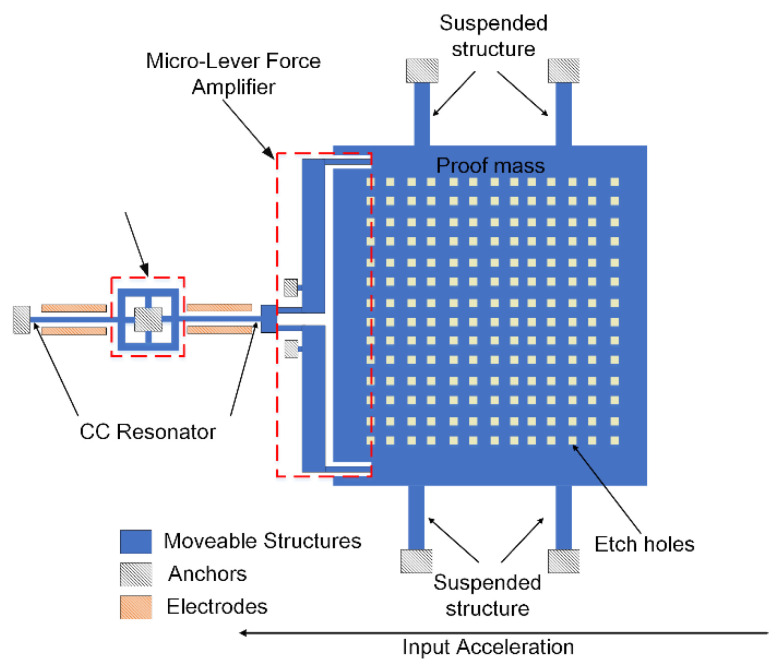
The schematic of the mode localized accelerometer.

The schematic of our mode localized accelerometer is shown in Figure 2. The two clamped-clamped (CC) resonators are coupled with each other by a micro-lever coupler. The two resonators are driven and sensed by parallel-plate capacitors at two sides of the resonators. The proof mass is suspended and connected to one of the CC resonators through a pair of micro-lever force amplifiers. When an acceleration is applied along the sensitivity axis direction, a corresponding perturbation is made through the micro-lever force amplifiers by the proof mass. The amplitude ratios of WCRs will change with the perturbation. 

The geometrical dimensions of the DETF-CC WCRs mode localized accelerometer are summarized in Table 1.

**Figure 3:**
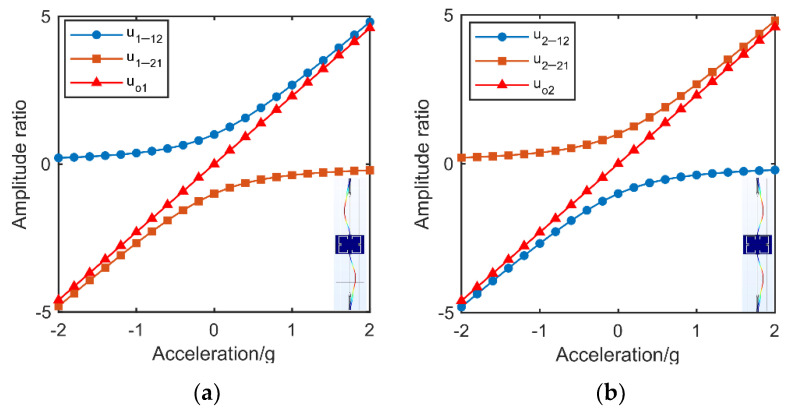
(**a**) Mode shape, amplitude ratios and linearity-enhanced output metric of the first mode. (**b**) Mode shape, amplitude ratios and linearity-enhanced output metric of the second mode.

We made verification of the linearity-enhanced output metric on our mode-localized accelerometer by taking a Finite Element Multiphysics (FEM) simulation. In the accelerometer, out-of-phase mode and in-phase mode were worked as the first mode (i = 1) and second mode (i = 2) as shown in Figure 3a,b, respectively. The amplitude ratios of the two modes and their linearity-enhanced output metric are shown in Figure 3a,b, respectively as well. 

## 3. Temperature Dependence Analysis of 2-DoF Accelerometer

### 3.1. The Dependence of Linearity-Enhanced Output Metric on Temperature

As the temperature changes, the difference in the thermal expansion coefficient between the materials and the residual stress generated in the fabrication process will cause an extra thermal perturbation on the WCRs besides the acceleration applied, inducing a bias drift with temperature on the output of mode-localized accelerometers. Furthermore, the coupling stiffness is also temperature dependent, especially in a mechanical coupling structure since the Young’s modulus of the silicon material will change with temperature. This effect will induce the sensitivity fluctuated with the temperature. Thus, the linearity-enhanced output metric (*i* = 1, 2) of a 2-DoF mode-localized accelerometer can be expressed as Equation (6) with considering of the impact caused by the temperature.

(6) $$u_{oi}\left(T\right)=\frac{\Delta k_{a}}{k_{c}\left(T\right)}+\frac{\Delta k_{T}\left(T\right)}{k_{c}\left(T\right)}$$

where $\Delta k_{a}$ is the perturbation caused by acceleration and $\Delta k_{T}\left(T\right)$ is the perturbation induced by internal thermal stress at the temperature T, while $k_{c}\left(T\right)$ is the coupling stiffness at temperature T.

### 3.2. The Dependence of Frequency on Temperature

The resonant frequencies of the two vibrational modes of a WCRs is also influenced by the temperature. Besides of the internal thermal stress and coupling stiffness, the changes in stiffness of the coupling resonators also make the resonant radian frequencies (i = 1, 2) temperature dependent as shown in Equation (7), where $\omega_{0}\left(T\right)=\sqrt{\frac{k\left(T\right)}{m}}$.

(7) $$\omega_{i}\left(T\right)=\left(\sqrt{1+\frac{1}{k\left(T\right)}(k_{c}\left(T\right)+\frac{\Delta k_{a}+\Delta k_{T}}{2}\mp \frac{1}{2}\sqrt{{\left(\Delta k_{a}+\Delta k_{T}\right)}^{2}+4k_{c}^{2}\left(T\right)}}\right)\omega_{0}\left(T\right)$$

## 4. The Method of Temperature Compensation

We took the linearity-enhanced output metric to measure the acceleration. The relationship on the linearity-enhanced output metrics and acceleration is expressed as (i = 1, 2):

(8) $$u_{oi}=S_{Fi}a+Bias_{i}$$

Since the sensitivity and bias against external stiffness perturbation of $u_{oi}$ i = 1, 2) are temperature dependent, the scale factor $S_{Fi}$ and bias $Bias_{i}$ against acceleration are also temperature dependent in a mode-localized accelerometer (i = 1, 2). Therefore, the main goal is to make compensation for $S_{Fi}$ and $Bias_{i}$ (i = 1, 2).

According to Equations (2)–(4), the relationship between resonant frequencies and amplitude ratios (i = 1, 2) can be derived as:

(9) $$\omega_{i}=\left[\sqrt{1+\frac{k_{c}\left(T\right)}{k\left(T\right)}(1+\frac{]}{u_{i-12}}})\right]\omega_{0}\left(T\right)=\left[\sqrt{1+\frac{k_{c}\left(T\right)}{k\left(T\right)}\left(1-u_{i-21}\right)}\right]\omega_{0}\left(T\right)$$

**Figure 4:**
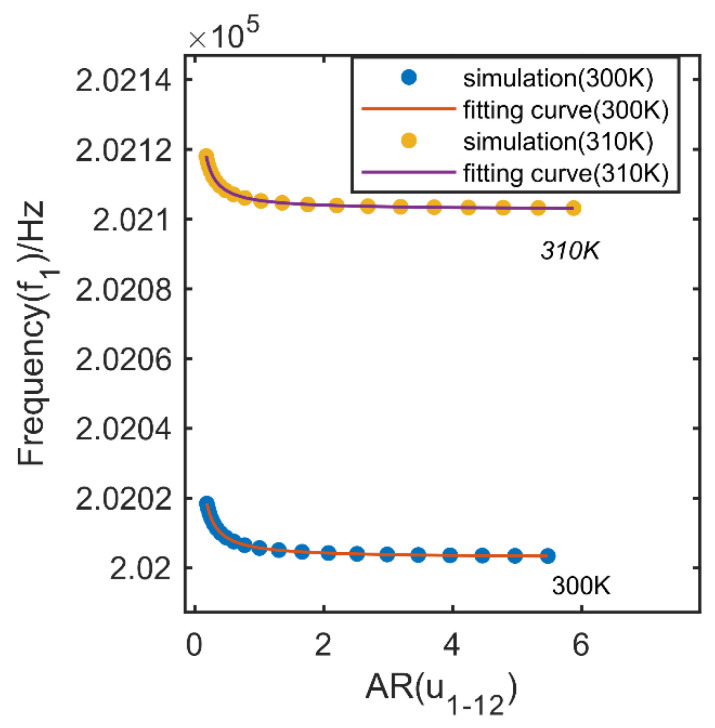
FEM simulation for the relationship between amplitude and resonant frequency under different temperature.

According to Equation (9), it is known that the relationship between resonant radian frequencies and amplitude ratios (i = 1, 2) is decided by the parameters of the coupling resonators which are dependent on the temperature. As the perturbation is consisted in the amplitude ratios and there is no term consisting of the perturbation made by acceleration applied, the relationship is independent on the applied acceleration. An FEM simulation was taken to prove the issues. As shown in Figure 4, by applying different acceleration over the range from −1 g to 1 g with a step of 0.1 g at 300 K and 310 K, respectively, simulation points were gotten and drawn in Figure 4. The fitting curves between resonant frequencies and amplitude ratios (i = 1) at 300 K and 310 K have the same format with Equation (9), with a coefficient of determination of $R^{2}=1$. Though the amplitude ratios changes with the acceleration applied, the relationship is fixed under a certain temperature.

By measuring resonant frequency $f_{i}$, amplitude ratios $u_{i-12}$ and $u_{i-21}$ (i = 1, 2) at real-time, the temperature T of the coupling resonators may be inferred with $\omega_{i}$ and $u_{i-12}$ or $u_{i-21}$ and then the scale factor $S_{Fi}$ and bias $Bias_{i}$ (i = 1, 2) by taking the linearity-enhanced output metric $u_{oi}$ (i = 1, 2) at T can be inferred, which can be formulated as (i = 1, 2)

(10) $$Bias_{i}\left(T\right)=G_{1}\left(f_{i},u_{i-12}\right)=G_{2}\left(f_{i},u_{i-21}\right)=G\left(f_{i},u_{i-12},u_{i-21}\right)$$

(11) $$S_{Fi}\left(T\right)=H_{1}\left(f_{i},u_{i-12}\right)=H_{2}\left(f_{i},u_{i-21}\right)=H\left(f_{i},u_{i-12},u_{i-21}\right)$$

By taking the linearity-enhanced output metric according to Equation (5) with Equation (8), the temperature compensated result of the accelerometer can be achieved as (*i* = 1, 2)

(12) $$a=\frac{u_{oi}-Bias_{i}\left(T\right)}{S_{Fi}\left(T\right)}$$

**Figure 5:**
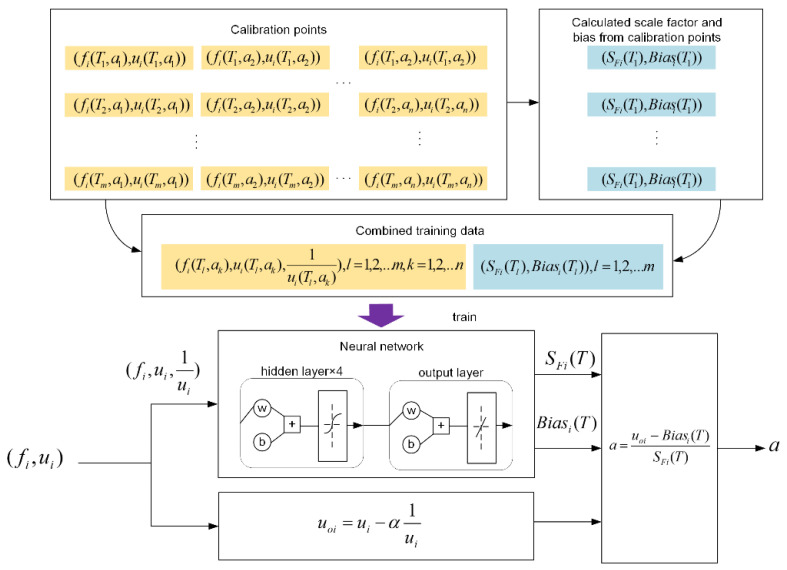
Data flow of the compensation process.

In this work, we use a five-layer feed-forward fitting neural network to explore G and H. The process of implementing proposed temperature compensation method consists of three steps. First, certain calibration points are measured. In this step, the amplitude ratio and resonant frequency of mode localized accelerometer with applied acceleration in full acceleration scales $\left(a_{1},a_{2},\ldots ,a_{n}\right)$ under different temperature $\left(T_{1},T_{2},\ldots ,T_{m}\right)$ over the full temperature range were recorded. In the second step, the scale factor $S_{Fi}$ and bias $Bias_{i}$ (i = 1, 2) under different temperature calculated from linearity-enhanced output metric are used to train the neural network together with the amplitude ratio and resonant frequency of corresponding temperature. In this network training process, the frequency, the amplitude ratio and its reciprocal at calibration temperature T work as network inputs and the scale factor, bias at temperature T work as desired output. Third, the trained neural network is used as the temperature model of the accelerometer and got the compensated result with Equation (12). A detailed data flow of the proposed method is shown in Figure 5.

## 5. Experiment

**Figure 6:**
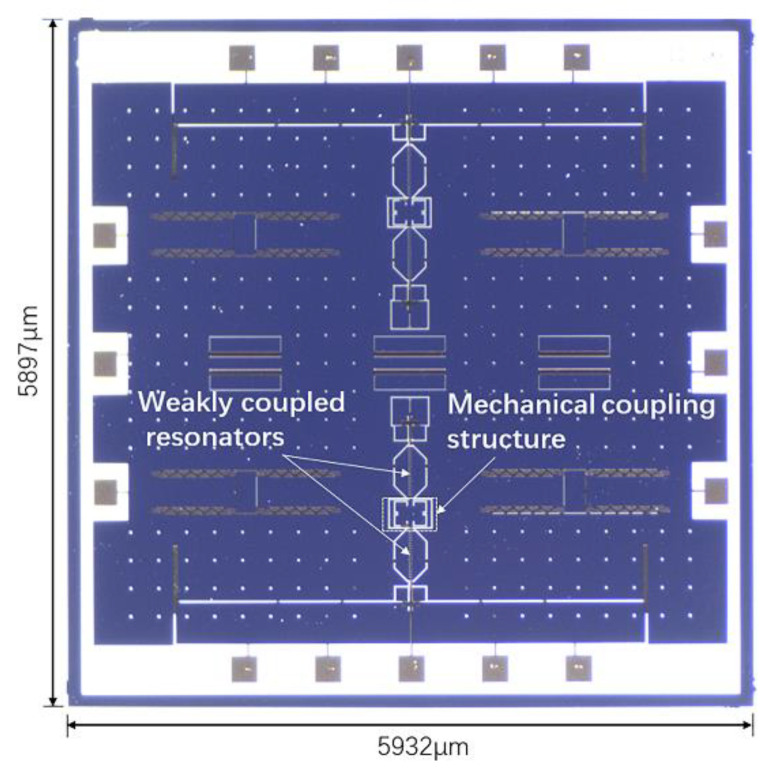
Photograph of mode-localized accelerometer.

We took experimental verification on our 2-DoF WCRs mode-localized accelerometer device. The accelerometer was fabricated by the silicon-on-glass (SOG) process. The photograph under optical microscope of the mode-localized accelerometer is shown in Figure 6.

**Figure 7:**
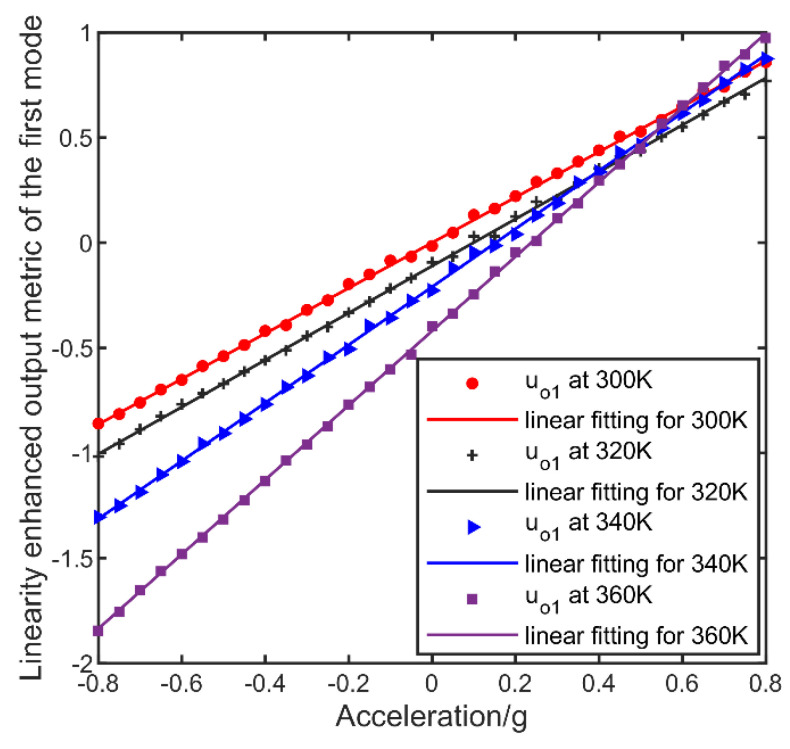
Data flow of the compensation process.

In our experiment, the mode-localized accelerometer worked in in-phase or the first mode. The linearity-enhanced output metric by Equation (5) where i = 1 was taken to measure the acceleration. Applied with different accelerations, the output metrics under different temperature were shown in Figure 7. As shown in Figure 7, with the temperature varied, the zero bias and scale factor changed from 0 g, 1.08 AR/g at 300 K to −0.42 g and 1.77 AR/g at 360 K, respectively. The zero bias and scale factor were proved to be temperature dependent which is corresponding with our analysis before.

**Figure 8:**
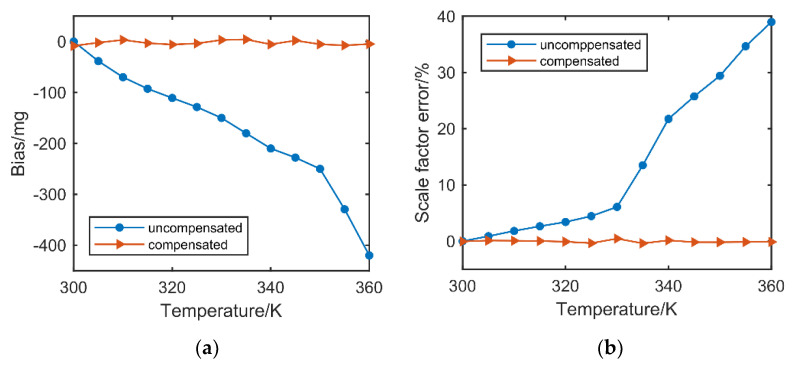
(**a**) Comparation on bias drifts of acceleration before and after compensation. (**b**) Comparation on errors of scale factor before and after compensation.

In the step of temperature compensation, we recorded the amplitude ratio $u_{1-12}$, resonant frequency with applied acceleration from −0.8 g to 0.8 g of 0.05 g step within the temperature range from 300 K to 360 K with a step of 5 K and made them as the dataset together with corresponding scale factor and bias for neural network. Then, the trained network was used to implement the compensation by the proposed method. The comparation before and after compensation was shown in Figure 8, where the bias acceleration and scale factor at 300 K were taken as the reference.

As shown in Figure 8a, the bias drift was decreased from 420 mg to less than 5.3 mg after compensation over the temperature range from 300 K to 360 K, which is about 79 times better than before compensation. And the scale factor error became less than 0.45% from 38.98% in the temperature range as shown in Figure 8b, achieving nearly 87 times of improvement in performance. We also made a comparison of our method with work [27] in Table 2, where work [27] decreased the influence of temperature by a good design on the structure. As shown in Table 2, both the performance in bias and scale factor against temperature were better in our work.

## 6. Conclusions

This article proposed a new method to compensate the affection of temperature fluctuation on 2-DoF mode-localized accelerometers. In the proposed method, the resonant frequency is applied to sensing temperature with the amplitude ratios of the coupled resonators itself, which may avoid the thermal lag. The method was proved to be effective and can be realized as a real-time temperature compensation for mode-localized accelerometers.

## Figures and Tables

**Table 1:** Parameters of the accelerometer.

Parameter	Value
Device thickness	40 μm
Length of CC resonant beam	400 μm
Width of CC resonant beam	7 μm
Gap of resonant beam	2 μm
Quality of proof mass	1.50 mg
Quality factor	15,600
Glass thickness	50 μm

**Table 2:** Comparison with work [27].

	This Work	[27]
Temperature range	300 K–360 K	303 K–333 K
Bias drift	0.088 mg/K	0.22 mg/K
Scale factor over the temperature range	0.45%	0.94%
